# Cognitive and affective perspective taking amongst adolescent offenders with variants of callous–unemotional traits

**DOI:** 10.1007/s00787-023-02356-9

**Published:** 2024-01-10

**Authors:** Stavroola A. S. Anderson, David J. Hawes, Pamela C. Snow

**Affiliations:** 1 School of Psychology, The University of Sydney, Sydney, NSW Australia; 2 School of Education, La Trobe University, Bendigo, VIC Australia

**Keywords:** Callous–unemotional traits, Cognitive empathy, Perspective taking, Youth offender, Antisocial behaviour

## Abstract

Evidence suggests that associations between antisocial behaviour, callous–unemotional (CU) traits and cognitive empathy (e.g. perspective taking) vary depending on more fine-grained dimensions of these constructs. This study examined associations between adolescent antisocial behaviour and individual differences in cognitive and affective perspective taking ability. Based on current theory regarding distinct variants of CU traits, we further tested whether the correlates of CU traits differed amongst youth with high versus low levels of anxiety. Participants were 130 male adolescents (81 youth offenders; 49 non-offenders) aged 13–20 years, of predominantly Caucasian and Aboriginal Australian ethnicity. Perspective taking skills were indexed using performance-based testing, and self-report data was collected on CU traits and anxiety in a cross-sectional design. Offender status was associated with poorer cognitive and affective perspective taking. In addition, associations between CU traits and perspective taking skills were moderated by anxiety. Specifically, CU traits were associated with poorer skills for second-order cognitive perspective taking amongst high-anxiety youth, whereas CU traits were associated with better cognitive and affective perspective taking skills amongst low-anxiety youth. More fine-grained assessment of such factors stands to enhance understanding of, and effective intervention for, antisocial youth.

## Cognitive and affective perspective taking amongst adolescent offenders with variants of callous–unemotional traits

The notion that antisocial individuals are characterised by deficits in empathy has a long history in the clinical and forensic literature; however, empirical evidence of this has often been inconsistent [1–3]. Recent advances in the conceptualization of empathy and antisocial behaviour have been informed extensively by models of heterogeneity amongst individuals with antisocial behaviour, particularly the distinction between those with high versus low levels of callous and unemotional (CU) traits. CU traits comprise features including a lack of remorse or guilt, deficient affect and lack of caring towards others, and have been conceptualised based on the affective component of psychopathy. A lack of empathy is considered core to psychopathy and CU traits, and accounts of this have emphasised a distinction between affective and cognitive constructs underlying empathy [4].

As conceptualised in current accounts (e.g. [5, 6]), affective components of empathy include processes such as affective sharing and physiological arousal to another’s emotion. Cognitive components include mentalising processes such as Theory of Mind (ToM), which refers to the capacity to understand others’ mental states; and perspective taking (PT), which refers to the recognition and understanding of another’s viewpoint based on situational cues. A further distinction has been made between cognitive PT and affective PT, the latter of which involves adopting another’s perspective to understand their emotional state. Current theory has further emphasised the hierarchical structure of these social-cognitive processes, differentiating predominantly cognitive mentalising processing, predominantly affective processes when witnessing others’ emotions, based on shared emotional, motor and somatosensory representations; and combined processes that engage cognitive and affective functions in parallel [7, 8].

Substantial neuroimaging research has implicated specific brain regions (e.g. medial prefrontal cortex, posterior cingulate cortex, precuneus and temporal parietal junction) in both cognitive and affective mentalising [9], and functional connectivity between these key brain regions is understood to develop across childhood to form a social network [10]. Evidence also suggests that specific brain regions are engaged during cognitive PT (e.g. dorsomedial and dorsolateral prefrontal cortex) as opposed to affective PT (e.g. ventromedial prefrontal cortex, amygdala and basal ganglia), and that cognitive PT appears more dependent on executive function [11]. Further, computational modeling has indicated that activation in the anterior cingulate cortex is associated with pro-social learning, and is stronger in individuals higher in empathy [12], who learn more quickly when benefitting others [13].

Youth with high levels of antisocial behaviour have been found to show disrupted connectivity between social network brain regions during social cognition tasks [14], as well as less efficient connectivity in frontoparietal regions supporting executive function [15]. Higher levels of conduct problems have also been found to be associated with delays in cognitive processes during pro-social decision making [16]. This evidence may explain why PT has been found to be significantly impaired amongst youth offenders compared to non-offenders [17], as well as amongst antisocial children [18].

Importantly, there is also extensive evidence that CU traits and psychopathy are associated with impairments in domains related to ToM and PT [19–21]. In terms of mechanisms that may account for this, youth with high levels of CU traits have been found to demonstrate irregular functional connectivity between social network brain regions including posterior cingulate cortex, precuneus, dorsomedial prefrontal cortex and amygdala (e.g. [22]). Higher CU traits have also been found to be associated with less functional connectivity between social network brain regions, which support perspective taking, and conflict network brain regions (e.g. anterior cingulate cortex, pre-supplementary motor area), linked to cognitive control [23], as well as information accumulation processes which support self-serving decisions during pro-social decision making [16].

Emerging evidence has, however, suggested that deficits in these cognitive components of empathy may be far more pronounced and complex than previously thought (for a review see [24]), both in adults with psychopathic traits (e.g. [25]), and children with CU traits (e.g. [26]). Evidence has further suggested that CU traits may be significantly associated with specific forms of PT in childhood and adolescence. In one key study, Anastassiou-Hadjicharalambos and Warden [27] found different associations for cognitive and affective PT in children. Their results revealed that, compared to typically social peers, antisocial children with low CU traits had deficits in both cognitive and affective PT, whilst those with high CU traits had selective deficits in affective PT. These findings are supported by evidence that conduct disordered children with high CU traits presented with deficits in affective PT [28] and that, amongst adolescents disengaged from school, CU traits were associated with a relative deficit in affective PT [29].

More recent evidence suggests that, for some individuals, CU traits could be associated with enhanced skills in cognitive PT. This evidence is based on the subtyping of primary and secondary variants of CU traits in terms of concurrent level of anxiety (primary variant, low anxiety; secondary variant, high anxiety) [30]. In novel research involving incarcerated youth offenders, Kahn et al. [31] investigated the moderating role of anxiety in associations between CU traits and empathy. Their results revealed no significant interactions between CU traits and anxiety in relation to either self-report or laboratory measures of affective empathy. However, findings demonstrated that CU traits and self-reported cognitive empathy were negatively correlated in individuals with high anxiety (i.e. secondary variant CU traits), but uncorrelated in individuals with low anxiety (i.e. primary variant CU traits). Further, these authors were able to assess different elements of cognitive empathy through a PT task, in which participants were required to interpret a character’s thought (cognitive PT) or feeling (affective PT). CU traits and cognitive PT were found to be positively correlated in individuals with low anxiety (primary variant CU traits), but not significantly correlated in individuals with high anxiety (secondary variant CU traits). In contrast, there were no significant interactions between CU traits and anxiety in relation to affective PT [31]. These potentially important yet unpredicted findings were highlighted as worthy of further investigation by Kahn et al. [31], as understanding the differences in empathy deficits between individuals with primary and secondary variants of CU traits could better inform individualised intervention for antisocial youth.

The major aim of the current study was to examine associations between PT, CU traits and youth offending. Moreover, attention was given to both cognitive and affective forms of PT, and to primary (high CU traits, low anxiety) and secondary (high CU traits, high anxiety) variants of CU traits. Although emerging evidence has been reported to suggest that youth with distinct variants of CU traits exhibit somewhat distinct deficits in cognitive PT (e.g. [31]), current conceptualisations of cognitive PT emphasise distinctions that have rarely been incorporated into such research to date. As such, the current study was designed to provide novel tests of these associations based on a more fine-grained examination of PT addressing both first- and second-order cognitive PT, as well as affective PT. In addition, there is a need for such research to index PT using ecologically valid measures that reflect real-world skills (as utilised by [18]), given the reliance on text, verbal and picture-based stimuli to date (e.g. [27, 31]). For this reason, in the current study, forms of PT were indexed using a video-based method involving conversational exchange in real-world social settings.

It was predicted, first, that antisocial behaviour would be associated with deficits in both cognitive and affective PT, such that youth offenders would have significantly poorer PT skills than non-offenders. The remaining hypotheses addressed associations between CU traits and PT based on CU variant and form of PT. Second, it was hypothesised that both primary and secondary variants of CU traits would be associated with poorer affective PT. Third, it was hypothesised that higher CU traits would be associated with better cognitive PT skills, but only amongst youth with primary variant CU traits. No such association was expected for youth with secondary variant CU traits or low CU traits.

## Method

### Participants

Participants were 130 male adolescents between the ages of 13 and 20 years (*M* = 16.32, *SD* = 1.35). Participants were included in the current research if they had undertaken most of their schooling in an English-speaking country, did not have a known diagnosis of intellectual impairment or hearing impairment, and were not known to be experiencing an acute episode of mental illness. Youth offenders (*n* = 81) were recruited through 19 youth justice centres (detention = 6; community service = 13), whilst non-offenders (*n* = 49) were recruited through five public secondary schools. All participants were resident in New South Wales, the most populous state in Australia. Most participants reported non-Indigenous Australian ethnicity (53.8%; majority Caucasian), but a substantial proportion reported Indigenous ethnicity (46.2%; majority Aboriginal). All participants reported that their primary language of communication was Standard Australian English. Using Socio-Economic Indexes for Areas [32], participants were assigned an Index of Relative Socio-Economic Advantage/Disadvantage (IRSAD; from one (lowest) to nine (highest)) based on postcode of usual residence. Participants had a mean IRSAD of 3.26, indicating relatively greater disadvantage and lack of advantage in general, consistent with lower socio-economic status (SES).

### Measures

Offender status was operationalised as a two-level categorical variable (youth offender; non-offender). A youth offender was categorised as such based on officially documented contact with a youth justice agency at the time of participation in research. This official contact could be in the form of either supervision through a youth justice community service or detention in a secure youth justice centre. Non-offender categorisation was based on self-report of no current or historical official contact with a youth justice agency.

CU traits were measured using the Inventory of Callous-Unemotional Traits (ICU; [33]). This self-report scale, based on restructuring of the Callous-Unemotional subscale of the Antisocial Process Screening Device (APSD; [34]), was designed to comprehensively assess the presence and magnitude of CU traits in youth. Participants were asked to respond to 24 items that were each rated on a 4-point scale (0 = not at all true, 1 = sometimes true, 2 = very true, and 3 = definitely true). However, given that items 2 and 10 have not shown consistently strong correlations with total scores in the self-report format [35], these items were excluded in calculating a 22-item-based total score. The validity of the 22-item ICU has been demonstrated in a range of research involving community (e.g. [36, 37]) and forensic (e.g. [35, 38]) samples of adolescents. In the current study, analysis revealed a Cronbach’s α score of 0.82 for the total 22-item scale, which was comparable to findings from previously referenced research (a range of 0.78–0.83).

Anxiety was measured using the Anxious-Depressed subscale of the Youth Self Report (YSR; [39]). The YSR is a questionnaire designed to assess adaptive and maladaptive functioning in adolescents, forms part of the Achenbach System of Empirically Based Assessment (ASEBA) and has been normed for ages 11–18 years. The Anxious-Depressed subscale consists of 16 items that are scored on a 3-point scale (0 = not true, 1 = somewhat true, 2 = very or often true). The YSR has demonstrated reliability and validity [39], and has been used extensively in research investigating psychosocial functioning in adolescents. Cronbach’s α reliabilities for the Anxious-Depressed subscale of the YSR in samples of adolescents, including samples of youth offenders, have been demonstrated to be high, ranging from 0.80 to 0.86 (e.g. [40–42]). In the current study, analysis revealed a Cronbach’s α 0.81 for the Anxious-Depressed subscale.

The Awareness of Social Inference Test (TASIT; [43]) was developed as an ecologically valid means to systematically assess different facets of social perception. TASIT has been designed for use with ages 13–60 years, and to differentiate between neurologically typical individuals and those with compromised skills. Participants were assessed using the Social Inference-Minimal Task, which involved viewing a series of 15 short, videotaped vignettes of actors interacting in everyday conversational exchanges. Five of these scenes represented sincere exchanges, where words and meaning were consistent, and ten represented sarcastic exchanges, in which paralinguistic cues indicate inconsistency between words and meaning. After watching each scene, participants were asked four questions, each capturing a distinct facet of the inferential process [44]. Two questions were representative of cognitive PT. ‘Belief’ questions examined the participants’ capacity to accurately construe what the speaker knew or believed, and represented first-order cognitive PT. ‘Intent’ questions examined the participants’ capacity to accurately construe what the speaker intended (including what they intended the listener to believe) and represented second-order cognitive PT. ‘Feel’ questions, representative of affective PT, examined the participants’ capacity to accurately construe what the speaker was feeling. ‘Say’ questions examined the participants’ capacity to accurately construe meaning from the conversational exchange but were not the focus of analysis in the current study. Participants were allocated one point for each correct response. Question category scores were then summed to produce total belief (first-order cognitive PT), intent (second-order cognitive PT) and feel (affective PT) scores.

### Procedure

The University of Sydney Human Research Ethics Committee, the Department of Communities and Justice, NSW, and the Department of Education, NSW, approved this research. Having received these approvals, inclusion criteria, participant information and consent forms were distributed to participating youth justice centres and schools. The first author visited all participating youth justice centres and schools to administer assessments and questionnaires on site. Testing commenced with a semi-structured interview followed by assessment of non-verbal ability with the Matrices subtest of the Kaufman Brief Intelligence Test, 2nd edition (KBIT-2) [45]. All other measures were presented in a random order. All items on the TASIT were read to participants, and demonstrations and practice opportunities were provided. Participants could choose to have items on the ICU and YSR read to them and have their responses recorded for them by the first author.

### Data analytic plan

All analyses were conducted using SPSS, Version 27 [46]. An a priori power analysis was conducted using G*Power 3.1 [47], based on recommendations by Dattalo [48], and considering use of a separate hierarchical regression analysis for each of three dependent variables. Based on the assumptions of an alpha of 0.05, a power of 0.8, and a medium effect size (Cohens *f*^2^ = 0.15), it was determined that the minimum desired sample size was 99.

Preliminary analyses revealed no violation of the assumptions of normality, linearity, multicollinearity and homoscedasticity. Hypotheses were tested using a set of three hierarchical regression analyses consisting of a separate model for each of the dependent variables of cognitive (first order; second order) and affective PT. All models included the same independent variables, which were entered in two blocks. Block one consisted of the continuous variables of age, SES, and non-verbal ability, and the recoded weighted categorical variable of ethnicity. Also entered in this block were the mean centred variables of CU traits and anxiety and the categorical variable of offender status (youth offender; non-offender). To test for associations with variants of CU traits based on high or low anxiety, the two-way interaction term for CU x anxiety was entered in a second block. To increase confidence in the statistical significance of coefficient terms, each hierarchical regression analysis was reanalysed on 2000 wild bootstrap samples [49] and the bootstrap distribution was corrected for bias and acceleration [50]. Significant interactions were probed using simple slope analyses involving tests of conditional effects of CU traits on each PT variable at high (1 standard deviation above the mean), medium (mean) and low (1 standard deviation below the mean) levels of anxiety, as described in Bauer and Curran [51].

## Results

### Descriptive statistics

Means, standard deviations and zero order correlations for relevant study variables are shown in Table 1. Status as a youth offender was associated with higher levels of CU traits (youth offender *M* = 25.41; non-offender *M* = 20.49) and anxiety (youth offender *M* = 6.23; non-offender *M* = 4.67). Status as a youth offender was also associated with poorer first-order cognitive PT (youth offender *M* = 11.70; non-offender *M* = 12.57), second-order cognitive PT (youth offender *M* = 10.98; non-offender *M* = 12.10) and affective PT (youth offender *M* = 12.79; non-offender *M* = 13.57). There were no significant correlations between CU traits or anxiety and either cognitive or affective PT. Age was significantly correlated with offence status and anxiety, with older age associated with status as a youth offender and slightly higher anxiety. Ethnicity was significantly correlated with both first- and second-order cognitive PT, but not affective PT. Non-Indigenous identification was associated with better cognitive PT. In contrast, higher SES was associated with better affective PT, but SES was not significantly correlated with cognitive PT. Non-verbal ability (NVA) was significantly correlated with offence status and PT, with higher NVA associated with status as a non-offender, and better cognitive and affective PT.

**Table 1:** Descriptive statistics and zero-order correlations

	*M*	*SD*	Correlation								
			1	2	3	4	5	6	7	8	9
1. Offence status	1.38	0.49									
2. CU traits	23.55	8.06	−0.30^***^								
3. Anxiety	5.65	4.21	−0.18^*^	−0.08							
4. PT Cog. 1st Ord	12.03	1.94	0.22^*^	0.02	0.03						
5. PT Cog. 2nd Ord	11.40	2.16	0.25^**^	−0.01	0.04	0.64^*^					
6. PT Aff	13.08	1.60	0.24^**^	0.07	0.06	0.59^*^	0.66^**^				
7. Age	16.38	1.36	−0.48^**^	0.15	0.18^*^	0.10	0.08	0.01			
8. Ethnicity	1.54	0.50	0.08	−0.12	0.03	0.23^**^	0.20^*^	0.07	−0.09		
9. SES	3.26	1.87	−0.19^*^	0.08	0.02	0.13	0.13	0.21^*^	0.06	0.03	
10. Non-verbal ability	92.40	13.67	0.43^**^	−0.06	0.08	0.28^**^	0.47^**^	0.33^**^	−0.12	0.09	0.10

*Notes*. ^***^ *p* ≤ 0.001; ^**^ *p* ≤ 0.01; ^*^ *p* ≤0 .05

*Variables:* offence status (based on official records; youth offender = 1, non-offender = 2); CU traits (ICU 22-item total scale score); Anxiety (YSR Anxious-Depressed subscale score); perspective taking (PT; TASIT, Social Inference Minimal): cognitive 1st order (Cog. 1st Ord.; sum of “Belief” scores); cognitive 2nd order (Cog. 2nd Ord.; sum of “Intent” scores); affective (Aff.; sum of “Feel” scores); age (at time of assessment, based on official records for date of birth); ethnicity (based on self-report of cultural identification; Indigenous = 1, non-Indigenous = 2); SES (in terms of SEIFA categories, based on self-report of most recent address); non-verbal ability (KBIT-2 Matrices subtest standard score)

### Tests of main study hypotheses

The results of hierarchical regression analyses testing main effects of offender status, CU traits and anxiety, as well as interactions between CU traits and anxiety, on cognitive and affective perspective taking appear in Table 2.

**Table 2:** Hierarchical regression analyses for cognitive and affective perspective taking with predictors offence status, CU traits and anxiety

With predictors CU traits, anxiety, offender status									
	Cognitive perspective taking						Affective perspective taking		
	First order			Second order					
	β	*b* [BCa 95% CI]	*R*^*2*^	β	*b* [BCa 95% CI]	*R*^*2*^	β	*b* [BCa 95% CI]	*R*^*2*^
Model 1									
Age	0.27^**^	0.38 [0.17, 0.57]		0.23^**^	0.36 [0.14, 0.60]		0.14	0.16 [−0.05, 0.37]	
Ethnicity	0.22^**^	0.86 [0.24, 1.52]		0.17^*^	0.74 [0.19, 1.31]		0.05	0.15 [−0.36, 0.62]	
SES	0.16	0.16 [0.01, 0.31]		0.11	0.13 [−0.05, 0.32]		0.23^**^	0.20 [0.07, 0.32]	
Non-verbal ability	0.14	0.02 [−0.00, 0.04]		0.38^***^	0.06 [0.04, 0.08]		0.18	0.02 [0.00, 0.04]	
Offender status	−0.33^**^	−1.33 [−2.15, −0.50]		−0.23^*^	−1.02 [−1.83, −0.23]		-−033^**^	−1.08 [−1.73, −0.45]	
ICU	0.10	0.02 [−0.01, 0.06]		0.06	0.02 [−0.03, 0.06]		0.15	0.03 [−0.00, 0.06]	
Anxiety	0.03	0.01 [0.05, 0.07]		0.01	0.01 [−0.06, 0.08]		0.09	0.03 [−0.02, 0.10]	
			0.21^***^			.31^***^			0.20^***^
Model 2									
ICU x Anxiety	−0.27^***^	−0.02 [−0.02, −0.01]		−0.35^***^	−0.02 [−0.03, −0.01]		−0.24^**^	−0.01 [−0.02, −0.01]	
			0.28^***^			.42^***^			0.26^**^

Notes: ^***^ *p* ≤ .001; ^**^ *p* ≤ .01; ^*^ *p* ≤ .05. β = standardised beta, *b* = unstandardized beta, BCa 95% CI = bias-corrected and accelerated 95% confidence interval (based on 2000 wild bootstrap samples)

Variables: age (centred); ethnicity (dichotomized with weighted effect size: Indigenous Australian = -.54; non-Indigenous Australian = .46); SES (centred); non-verbal ability (KBIT-2 Matrices subtest standard score; centred); offender status (dichotomized with weighted effect coding: youth offender = 0.38; non-offender = -0.62); CU traits (ICU 22-item total scale score; centred); anxiety (YSR Anxious-Depressed subscale score; centred); perspective taking (TASIT, Social Inference Minimal): cognitive 1st order (sum of “belief” scores); cognitive 2nd order (sum of “Intent” scores); affective (sum of “Feel” scores)

**Fig. 1:**
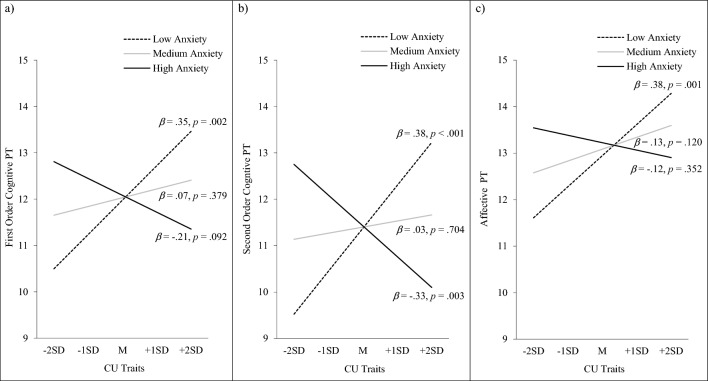
Associations between perspective taking and CU traits amongst youth with high versus low levels of anxiety. (**a**) First-order cognitive PT. (**b**) Second-order cognitive PT. (**c**) Affective PT

In the model testing predictors of first-order cognitive PT, there was a significant main effect for offender status (ß = −0.33, *p* = 0.003), in which status as an offender was associated with poorer first-order cognitive PT. There were no significant main effects for CU traits or anxiety. However, there was a significant interaction between CU traits and anxiety in this model (ß = −0.27, *p* < 0.001). This significant interaction was probed by testing the conditional effects of CU traits on first-order cognitive PT at high and low anxiety (as illustrated in Fig. 1a). CU traits were significantly related to first-order cognitive PT when anxiety was low (ß = 0.35, *p* = 0.002), but not when anxiety was high. That is, higher scores on CU traits were associated with better first-order cognitive PT, but only for youth low in anxiety (i.e. primary variant CU traits). For youth high in anxiety (i.e. secondary variant CU traits), there were no significant associations between CU traits and first-order cognitive PT.

In the model testing predictors of second-order cognitive PT, there was a significant main effect for offender status (ß = −0.23, *p* = 0.029), in which status as an offender was associated with poorer second-order cognitive PT. There were no significant main effects for CU traits or anxiety. However, there was a significant interaction between CU traits and anxiety in this model (ß = −0.35, *p* < 0.001). This significant interaction was probed by testing the conditional effects of CU traits on second-order cognitive PT at high and low anxiety (as illustrated in Fig. 1b). CU traits were significantly related to second-order cognitive PT when anxiety was low (ß = 0.38, *p* < 0.001), as well as when anxiety was high (ß = −0.33, *p* = 0.003). That is, higher scores on CU traits were associated with better second-order cognitive PT for youth low in anxiety (i.e. primary variant CU traits), but with poorer second-order cognitive PT for youth high in anxiety (i.e. secondary variant CU traits).

In the model testing predictors of affective PT, there was a significant main effect for offender status (ß = −0.33, *p* = 0.004), in which status as an offender was associated with poorer affective PT. There were no significant main effects for CU traits or anxiety. However, there was a significant interaction between CU traits and anxiety in this model (ß = −0.24, *p* = 0.004). This significant interaction was probed by testing the conditional effects of CU traits on affective PT at high and low anxiety (as illustrated in Fig. 1c). CU traits were significantly related to affective PT when anxiety was low (ß = 0.38, *p* = 0.001), but not when anxiety was high. That is, higher scores on CU traits were associated with better affective PT, but only for youth low in anxiety (i.e. primary variant CU traits). For youth high in anxiety (i.e. secondary variant CU traits), there were no significant associations between CU traits and affective PT.

## Discussion

The current study investigated associations between antisocial behaviour, CU traits and cognitive and affective perspective taking. Findings support the hypothesis that associations between CU traits and cognitive PT are moderated by anxiety and may, therefore, differ between putative variants of CU traits. Specifically, amongst low-anxiety youth, higher levels of CU traits were associated with better cognitive PT skills (both first and second order). This finding is consistent with Kahn et al. [31] who found that CU traits were associated with better cognitive PT, but only amongst adolescents with lower levels of anxiety. Amongst high-anxiety youth, there was no association between these CU traits and first-order cognitive PT. Amongst high-anxiety youth, higher CU traits were, however, associated with poorer second-order cognitive PT. Considerable research has previously tested, and supported the associations between CU traits and multiple components of empathy (e.g. PT, ToM), independent of anxiety-based variants. Our findings build on this previous work by suggesting that these associations may further vary according to type of variant, as well as type of PT. They suggest that associations between CU traits and cognitive PT vary both according to level of anxiety, and according to type of cognitive PT (first- and second-order forms). This may explain why our results diverge somewhat from research based on more global indices of PT, such as previous findings of a non-significant negative association between CU traits and overall cognitive PT in individuals with high levels of anxiety [31].

The current findings are also consistent with broader conceptualisations of primary and secondary variant CU traits. Amongst high-anxiety youth in the current sample, CU traits were associated with poorer skills for inferring what a speaker intended another to believe (second-order cognitive PT), suggesting deficits in more complex cognitive PT skills. This is consistent with previous findings for antisocial children [18], and may be indicative of reduced connectivity amongst neural social networks found in youth with elevated CU traits [22]. It is also noteworthy that the secondary variant of CU traits is associated with more severe childhood maltreatment [52–54], which has been linked to delayed development of higher level theory of mind [55–57], as well as decreased functional connectivity between key brain regions [58]. In contrast, amongst low-anxiety individuals in the current sample, CU traits were associated with enhanced ability to infer both what a speaker believed and what a speaker intended another to believe.

As noted by Kahn et al. [31], the notion that primary variant CU traits may be associated with better cognitive perspective taking can be seen as consistent with theories and past research suggesting that individuals with these traits have an enhanced ability to notice when others are vulnerable, which may facilitate self-serving manipulative behaviour towards others [59–62]. Not only was this form of cognitive empathy specifically associated with CU traits amongst low-anxiety youth in our sample, but so too was affective PT—the other major form of cognitive empathy indexed. It is noteworthy that the relationship between affective PT and CU traits was previously not found to be moderated by anxiety in the only study that has investigated this specific association to date [31]. It is unclear whether methodological differences in sampling or measurement in the current study may account for the mixed findings between these studies. It is important to note that TASIT places demands on complex social inference by requiring participants to process cues in social interactions that specifically involve sarcasm. Recent evidence suggests that high levels of psychopathic traits are more strongly associated with use of sarcasm and associated aggressive humour styles than high levels of other “dark triad” personality traits [63]. Also, it has been suggested that individuals high in psychopathy exhibit difficulties in affective ToM which are relative to the complexity of evaluation required and associated demand on cognitive resources [62]. In light of this, it is conceivable that greater attunement to sarcastic communication forms may have limited the cognitive demands involved in processing affective PT amongst those higher in primary CU traits in the current study.

The method used to measure PT in the current study may also potentially account for there being no significant relationship found between overall CU traits (i.e. not distinguished by level of anxiety) and the PT variables. The instructions for TASIT serve to cue participants to attend to the information in the PT vignettes that pertain to the follow-up questions, and inform participants that these questions are presented in a set structure for each vignette. There is some evidence that individuals high in CU or psychopathic traits are capable of engaging in both cognitive PT [64] and affective PT [65] when provided with such goal directed attentional instructions. In addition, TASIT involves a particularly real-world test of PT comprising video-based interpersonal cues [43]. There is evidence that similar video-based social cognition tasks co-activate brain regions associated with both cognitive and affective processes (e.g. [66]), which may facilitate engagement of more neurocognitive resources [8]. In contrast, previous research has often indexed PT using tasks that activate brain regions associated with cognitive processes specifically [8]. Further, whilst TASIT vignettes include representations of both basic (e.g. happy, angry) and complex (e.g. annoyed, impressed) emotions, the response format for affective PT involved naming a single emotion and nominating the presence or absence of that emotion. Higher CU traits have been shown to be associated with a greater deficit in recognising complex emotions than basic emotions [67], which is amplified when increased demands are placed on cognitive control [68]. However, in these studies, the task (i.e. Reading the Mind in the Eyes) featured a potentially more complex response format than TASIT, requiring participants to select the appropriate emotion from a list of four options. Various elements of TASIT, including instructions, stimuli and response format, may have, therefore, inadvertently supported high-CU participants to engage effectively in PT through provision of attentional cues, prompting engagement of neurocognitive resources and minimising task complexity.

In terms of associations between antisocial behaviour and perspective taking, participant status as an offender was associated with both significantly poorer cognitive PT (first and second order) and significantly poorer affective PT. This is consistent with predictions, and with previous research that has found adolescent and adult offenders to have poor perspective taking skills in general [17, 69, 70]. Current findings are also consistent with evidence that individuals high in antisocial behaviour show disrupted neural connectivity in regions supporting executive function when engaged in socio-emotional processing, which may impede information processing and decision making [15].

The current findings have two key implications for intervention and service provision for antisocial youth. First, the current findings highlight the importance of examining risk and protective factors for antisocial behaviour at a fine-grained level. Specifically, current evidence underscores the contribution assessment of anxiety and elements of empathy could bring to understanding both the developmental histories and prospects of antisocial individuals with high levels of CU traits. This is particularly important for youth engaged with the justice system, where the risk-need-responsivity model [71] and risk assessment inventories (e.g. [72]) influence both the level of supervision and type of intervention an individual is likely to receive in response to their criminal behaviour [73]. Second, it is possible that by adolescence, individuals with primary variant CU traits may have already begun to adapt PT skills for antisocial purposes, whilst for individuals with secondary variant CU traits, poorer PT skills may have increased vulnerability to engagement in antisocial behaviour. Earlier identification could, at the very least, provide opportunities for development of core skills (such as perspective taking) that might facilitate more appropriate socialisation (e.g. [52, 74]), with specific strategies for intervention informed by evidence of patterns of neurocognitive activation during pro-social learning [68].

When interpreting the results reported for this study, several limitations must be taken into consideration. Given that analyses did not control for the severity of conduct problems, which is known to covary with CU traits, we cannot infer that the results are attributable to CU traits alone. Moreover, although age was included as a covariate, we did not control for pubertal development, which has previously been associated with both CU traits and ToM. The current sample included only male participants. It is important that findings are replicated with female participants because there is evidence that different associations exist between CU traits and anxiety [26], empathy [75] and antisocial behaviour [76] in females, as well as profiles of cognitive processes that are distinct for males [16]. In addition, as this was a cross-sectional study, it is essential that the results are not interpreted in terms of causal mechanisms. Ideally, the findings from the current and related (e.g. [29, 31]) studies will inform future longitudinal investigations of the temporal development of different facets of cognitive and affective PT amongst youth at high risk for developing primary or secondary variants of CU traits.

Although multiple forms of PT were examined in the current study, these were nonetheless indexed by a single test. In future research, assessment made up of several tests of these capacities could provide more detailed evidence of the differences in PT skill profiles (e.g. [18, 77]) between individuals exhibiting primary and secondary variants of CU traits. Also, analysis of composite measures of multiple tests may potentially provide more robust support for the emerging evidence that primary variant CU traits may be associated with better PT ability. Finally, it should be noted also that whilst our analytic plan was informed by that of Kahn et al. [31], person-centred clustering approaches such as latent class analysis may offer further advantages, and should be considered in future research.

The findings of the current study highlight the value of applying a fine-grained conceptualisation to several developmental factors implicated in current models of antisocial behaviour. Our findings support the distinction between primary and secondary variants of adolescent CU traits based on level of anxiety, which in our sample were associated with significantly different skill profiles for perspective taking. This may in turn indicate that these subgroups of youth may benefit from distinct intervention. Our findings may also inform models of the developmental mechanisms that contribute to antisocial outcomes and CU traits across childhood and adolescence. That anxiety moderated associations between CU traits and higher levels of cognitive and affective PT skills in the manner seen here suggests that perception of the beliefs, intentions and feelings of others may facilitate manipulative antisocial behaviour in individuals with primary variant CU traits. Whilst literature on psychopathy and CU traits has generally emphasised deficits in empathy, it has become apparent that this is not universal to all manifestations of psychopathy or CU traits, or all forms of empathy. As has been demonstrated in the current study, a key strategy in developing a greater understanding of these associations is to employ a nuanced perspective.

## Data Availability

The data that support the findings of this study are not openly available due to reasons of sensitivity and are available from the corresponding author upon reasonable request.

## References

[1] Farrington DP, Jolliffe D (2021) Empathy, convictions, and self-reported offending of males and females in the Cambridge study in delinquent development. In: Empathy versus Offending, Aggression, and Bullying. Routledge, p 77–88

[2] Lovett BJ, Sheffield RA (2007) Affective empathy deficits in aggressive children and adolescents: a critical review. Clin Psychol Rev 27:1–13. 10.1016/j.cpr.2006.03.003 16697094 10.1016/j.cpr.2006.03.003

[3] Vachon DD, Lynam DR, Johnson JA (2014) The (non) relation between empathy and aggression: surprising results from a meta-analysis. Psychol Bull 140:751. 10.1037/a0035236 24364745 10.1037/a0035236

[4] Frick PJ, Kemp EC (2021) Conduct disorders and empathy development. Annu Rev Clin Psychol 17:391–416. 10.1146/annurev-clinpsy-081219-105809 33290109 10.1146/annurev-clinpsy-081219-105809

[5] de Waal FBM, Preston SD (2017) Mammalian empathy: behavioural manifestations and neural basis. Nat Rev Neurosci 18:498–509. 10.1038/nrn.2017.72 28655877 10.1038/nrn.2017.72

[6] Decety J, Holvoet C (2021) The emergence of empathy: a developmental neuroscience perspective. Dev Rev 62:100999. 10.1016/j.dr.2021.100999 10.1016/j.dr.2021.100999

[7] Maliske LZ, Schurz M, Kanske P (2023) Interactions within the social brain: co-activation and connectivity among networks enabling empathy and theory of mind. Neurosci Biobehav Rev 147:105080. 10.1016/j.neubiorev.2023.105080 36764638 10.1016/j.neubiorev.2023.105080

[8] Schurz M, Radua J, Tholen MG, Maliske L, Margulies DS, Mars RB, Sallet J, Kanske P (2021) Toward a hierarchical model of social cognition: a neuroimaging meta-analysis and integrative review of empathy and theory of mind. Psychol Bull 147:293–327. 10.1037/bul0000303 33151703 10.1037/bul0000303

[9] Fehlbaum LV, Borbás R, Paul K, Eickhoff SB, Raschle NM (2022) Early and late neural correlates of mentalizing: ALE meta-analyses in adults, children and adolescents. Soc Cogn Affect Neurosci 17:351–366. 10.1093/scan/nsab105 34545389 10.1093/scan/nsab105 PMC8972312

[10] McCormick EM, van Hoorn J, Cohen JR, Telzer EH (2018) Functional connectivity in the social brain across childhood and adolescence. Soc Cogn Affect Neurosci 13:819–830. 10.1093/scan/nsy064 30085317 10.1093/scan/nsy064 PMC6123525

[11] Healey ML, Grossman M (2018) Cognitive and affective perspective-taking: evidence for shared and dissociable anatomical substrates. Front Neurol 9:491. 10.3389/fneur.2018.00491 29988515 10.3389/fneur.2018.00491 PMC6026651

[12] Lockwood PL, Wittmann MK, Nili H, Matsumoto-Ryan M, Abdurahman A, Cutler J, Husain M, Apps MAJ (2022) Distinct neural representations for prosocial and self-benefiting effort. Curr Biol 32:4172.e4177-4185.e4177. 10.1016/j.cub.2022.08.01 36029773 10.1016/j.cub.2022.08.01 PMC9616728

[13] Lockwood PL, Apps MAJ, Valton V, Viding E, Roiser JP (2016) Neurocomputational mechanisms of prosocial learning and links to empathy. Proc Natl Acad Sci 113:9763–9768. 10.1073/pnas.1603198113 27528669 10.1073/pnas.1603198113 PMC5024617

[14] Dugré JR, Radua J, Carignan-Allard M, Dumais A, Rubia K, Potvin S (2020) Neurofunctional abnormalities in antisocial spectrum: a meta-analysis of fMRI studies on five distinct neurocognitive research domains. Neurosci Biobehav Rev 119:168–183. 10.1016/j.neubiorev.2020.09.013 32956690 10.1016/j.neubiorev.2020.09.013

[15] Tillem S, Dotterer HL, Goetschius LG, Lopez-Duran N, Mitchell C, Monk CS, Hyde LW (2023) Antisocial behavior is associated with reduced frontoparietal network efficiency in youth. Soc Cogn Affect Neurosci 18:nsad026. 10.1093/scan/nsad026 37148314 10.1093/scan/nsad026 PMC10275549

[16] Winters DE, Pettine WW, Sakai JT (2023) Cognitive mechanisms underlying prosocial decision making in callous-unemotional traits. J Psychopathol Behav Assess 45:308–321. 10.1007/s10862-023-10043-x 37608928 10.1007/s10862-023-10043-x PMC10441623

[17] Morosan L, Badoud D, Zaharia A, Brosch T, Eliez S, Bateman A, Heller P, Debbané M (2017) Emotion recognition and perspective taking: a comparison between typical and incarcerated male adolescents. PLoS ONE 12:e0170646. 10.1371/journal.pone.0170646 28122048 10.1371/journal.pone.0170646 PMC5266284

[18] Roberts R, McCrory E, Bird G, Sharp M, Roberts L, Viding E (2020) Thinking about others’ minds: mental state inference in boys with conduct problems and callous-unemotional traits. J Abnorm Child Psychol 48:1279–1290. 10.1007/s10802-020-00664-1 32632744 10.1007/s10802-020-00664-1 PMC7445196

[19] Chang S-AA, Tillem S, Benson-Williams C, Baskin-Sommers A (2021) Cognitive empathy in subtypes of antisocial individuals. Front Psychiatry 12:1070. 10.3389/fpsyt.2021.677975 10.3389/fpsyt.2021.677975 PMC8287099 34290630

[20] Ritchie MB, Neufeld RWJ, Yoon M, Li A, Mitchell DGV (2022) Predicting youth aggression with empathy and callous unemotional traits: a Meta-analytic review. Clin Psychol Rev 98:102186. 10.1016/j.cpr.2022.102186 36240695 10.1016/j.cpr.2022.102186

[21] Waller R, Wagner NJ, Barstead MG, Subar A, Petersen JL, Hyde JS, Hyde LW (2020) A meta-analysis of the associations between callous-unemotional traits and empathy, prosociality, and guilt. Clin Psychol Rev 75:101809. 10.1016/j.cpr.2019.101809 31862383 10.1016/j.cpr.2019.101809

[22] Winters DE, Hyde LW (2022) Associated functional network connectivity between callous-unemotionality and cognitive and affective empathy. J Affect Disord 318:304–313. 10.1016/j.jad.2022.08.103 36063973 10.1016/j.jad.2022.08.103 PMC10039983

[23] Winters DE, Leopold DR, Carter RM, Sakai JT (2023) Resting-state connectivity underlying cognitive control’s association with perspective taking in callous-unemotional traits. Psychiatry Res Neuroimaging 331:111615. 10.1016/j.pscychresns.2023.111615 36924739 10.1016/j.pscychresns.2023.111615 PMC10133184

[24] Campos C, Pasion R, Azeredo A, Ramião E, Mazer P, Macedo I, Barbosa F (2022) Refining the link between psychopathy, antisocial behavior, and empathy: a meta-analytical approach across different conceptual frameworks. Clin Psychol Rev 94:102145. 10.1016/j.cpr.2022.102145 35349788 10.1016/j.cpr.2022.102145

[25] Deming P, Dargis M, Haas BW, Brook M, Decety J, Harenski C, Kiehl KA, Koenigs M, Kosson DS (2020) Psychopathy is associated with fear-specific reductions in neural activity during affective perspective-taking. Neuroimage 223:117342. 10.1016/j.neuroimage.2020.117342 32898678 10.1016/j.neuroimage.2020.117342 PMC9831240

[26] Dadds MR, Hawes DJ, Frost ADJ, Vassallo S, Pl B, Hunter K, Merz S (2009) Learning to ‘talk the talk’: the relationship of psychopathic traits to deficits in empathy across childhood. J Child Psychol Psychiatry 50:599–606. 10.1111/j.1469-7610.2008.02058.x 19445007 10.1111/j.1469-7610.2008.02058.x

[27] Anastassiou-Hadjicharalambous X, Warden D (2008) Cognitive and affective perspective-taking in conduct-disordered children high and low on callous-unemotional traits. Child Adolesc Psychiatry Ment Health 2:16. 10.1016/j.psychres.2013.08.033 18601753 10.1016/j.psychres.2013.08.033 PMC2500005

[28] O’Kearney R, Salmon K, Liwag M, Fortune C-A, Dawel A (2017) Emotional abilities in children with oppositional defiant disorder (ODD): impairments in perspective-taking and understanding mixed emotions are associated with high callous–unemotional traits. Child Psychiatry Hum Dev 48:346–357. 10.1007/s10578-016-0645-4 27100725 10.1007/s10578-016-0645-4

[29] Lui JHL, Barry CT, Sacco DF (2016) Callous-unemotional traits and empathy deficits: mediating effects of affective perspective-taking and facial emotion recognition. Cogn Emot 30:1049–1062. 10.1080/02699931.2015.1047327 26192073 10.1080/02699931.2015.1047327

[30] Craig SG, Goulter N, Moretti MM (2021) A systematic review of primary and secondary callous-unemotional traits and psychopathy variants in youth. Clin Child Fam Psychol Rev 24:65–91. 10.1007/s10567-020-00329-x 33079293 10.1007/s10567-020-00329-x

[31] Kahn RE, Frick PJ, Golmaryami FN, Marsee MA (2017) The moderating role of anxiety in the associations of callous-unemotional traits with self-report and laboratory measures of affective and cognitive empathy. J Abnorm Child Psychol 45:583–596. 10.1007/s10802-016-0179-z 27364345 10.1007/s10802-016-0179-z

[32] Adhikari P (2006) Socio-economic indexes for areas: introduction, use and future directions. Australian Bureau of Statistics, Canberra

[33] Frick PJ (2003) The inventory of callous-unemotional traits. In: Unpublished rating scale. The University of New Orleans

[34] Frick PJ, Hare RD (2001) The antisocial process screening device. Multi-Health Systems, Toronto

[35] Kimonis ER, Frick PJ, Skeem JL, Marsee MA, Cruise K, Munoz LC, Aucoin KJ, Morris AS (2008) Assessing callous–unemotional traits in adolescent offenders: validation of the Inventory of Callous-Unemotional Traits. Int J Law Psychiatry 31:241–252. 10.1016/j.ijlp.2008.04.002 18514315 10.1016/j.ijlp.2008.04.002

[36] Docherty M, Boxer P, Huesmann LR, O’Brien M, Bushman B (2017) Assessing callous-unemotional traits in adolescents: determining cutoff scores for the inventory of callous and unemotional traits. J Clin Psychol 73:257–278. 10.1002/jclp.22313 27196809 10.1002/jclp.22313 PMC7219563

[37] Kemp EC, Ray JV, Frick PJ, Robertson EL, Fanti KA, Essau CA, Baroncelli A, Ciucci E, Bijttebier P (2022) Inventory of Callous-Unemotional Traits (ICU) factor structure and measurement invariance in an adolescent multinational sample. J Clin Child Adolesc Psychol:1–12. 10.1080/15374416.2022.2148531 10.1080/15374416.2022.2148531 36450005

[38] Ray JV, Frick PJ, Thornton LC, Steinberg L, Cauffman E (2016) Positive and negative item wording and its influence on the assessment of callous-unemotional traits. Psychol Assess 28:394–404. 10.1037/pas0000183 26121386 10.1037/pas0000183

[39] Achenbach TM, Rescorla LA (2001) Manual for the ASEBA school-age forms and profiles. University of Vermont, Research Center for Children, Youth, & Families, Burlington

[40] Breuk RE, Clauser CAC, Stams GJJM, Slot NW, Doreleijers TAH (2007) The validity of questionnaire self-report of psychopathology and parent–child relationship quality in juvenile delinquents with psychiatric disorders. J Adolesc 30:761–771. 10.1016/j.adolescence.2006.10.003 17161456 10.1016/j.adolescence.2006.10.003

[41] Kimonis ER, Tatar JR, Cauffman E (2012) Substance related disorders among juvenile offenders: what role do psychopathic traits play? Psychol Addict Behav 26:212–225. 10.1037/a0028047 22564205 10.1037/a0028047 PMC3374029

[42] Van Meter A, Youngstrom E, Youngstrom JK, Ollendick T, Demeter C, Findling RL (2014) Clinical decision making about child and adolescent anxiety disorders using the Achenbach System of Empirically Based Assessment. J Clin Child Adolesc Psychol 43:552–565. 10.1080/15374416.2014.883930 24697608 10.1080/15374416.2014.883930 PMC4101065

[43] McDonald S, Flanagan S, Rollins J (2011) The awareness of social inference test – revised. Pearson Assessment, Sydney

[44] McDonald S, Flanagan S, Rollins J, Kinch J (2003) TASIT: aanew clinical tool for assessing social perception after traumatic brain injury. J Head Trauma Rehabil 18:219–238. 10.1097/00001199-200305000-00001 12802165 10.1097/00001199-200305000-00001

[45] Kaufman AS, Kaufman NL (1990) Kaufman brief intelligence test. American Guidance Service, Circle Pines

[46] IBM Corp (2020) IBM SPSS statistics for windows, version 27.0. IBM Corp., Armonk

[47] Faul F, Erdfelder E, Lang A-G, Buchner A (2007) G*Power 3: a flexible statistical power analysis program for the social, behavioral, and biomedical sciences. Behav Res Methods 39:175–191. 10.3758/BF03193146 17695343 10.3758/BF03193146

[48] Dattalo P (2018) Determining sample size using fast and slow thinking. J Soc Serv Rese 44:180–190. 10.1080/01488376.2018.1436632 10.1080/01488376.2018.1436632

[49] Stute W, Manteiga WG, Quindimil MP (1998) Bootstrap approximations in model checks for regression. J Am Stat Assoc 93:141–149. 10.2307/2669611 10.2307/2669611

[50] Efron B (1987) Better bootstrap confidence intervals. J Am Stat Assoc 82:171–185. 10.1080/01621459.1987.10478410 10.1080/01621459.1987.10478410

[51] Bauer DJ, Curran PJ (2005) Probing interactions in fixed and multilevel regression: inferential and graphical techniques. Multivariate Behav Res 40:373–400. 10.1207/s15327906mbr4003_5 26794689 10.1207/s15327906mbr4003_5

[52] Cecil CAM, McCrory EJ, Barker ED, Guiney J, Viding E (2018) Characterising youth with callous–unemotional traits and concurrent anxiety: evidence for a high-risk clinical group. Eur Child Adolesc Psychiatry 27:885–898 29222633 10.1007/s00787-017-1086-8 PMC6013514

[53] Kahn RE, Frick PJ, Youngstrom EA, Kogos Youngstrom J, Feeny NC, Findling RL (2013) Distinguishing primary and secondary variants of callous-unemotional traits among adolescents in a clinic-referred sample. Psychol Assess 25:966–978. 10.1037/b0000003 23647031 10.1037/b0000003 PMC3902637

[54] Kimonis ER, Frick PJ, Cauffman E, Goldweber A, Skeem J (2012) Primary and secondary variants of juvenile psychopathy differ in emotional processing. Dev Psychopathol 24:1091–1103. 10.1017/S0954579412000557 22781873 10.1017/S0954579412000557

[55] Cicchetti D, Rogosch FA, Maughan A, Toth SL, Bruce J (2003) False belief understanding in maltreated children. Dev Psychopathol 15:1067–1091. 10.1017/S0954579403000440 14984138 10.1017/S0954579403000440

[56] O’Reilly J, Peterson CC (2015) Maltreatment and advanced theory of mind development in school-aged children. J Fam Violence 30:93–102. 10.1007/s10896-014-9647-9 10.1007/s10896-014-9647-9

[57] Pears KC, Fisher PA (2005) Emotion understanding and theory of mind among maltreated children in foster care: evidence of deficits. Dev Psychopathol 17:47–65. 10.1017/S0954579405050030 15971759 10.1017/S0954579405050030

[58] Teicher MH, Samson JA, Anderson CM, Ohashi K (2016) The effects of childhood maltreatment on brain structure, function and connectivity. Nat Rev Neurosci 17:652–666. 10.1038/nrn.2016.111 27640984 10.1038/nrn.2016.111

[59] Cleckley H (1941) The mask of sanity: an attempt to reinterpret the so-called psychopathic personality. The C. V. Mosby company, St. Louis

[60] Salekin RT, Andershed H, Clark AP (2018) Psychopathy in children and adolescents: assessment and critical questions regarding conceptualization. In: Patrick CJ (ed) Handbook of psychopathy. The Guilford Press, New York, pp 479–508

[61] Skeem JL, Poythress N, Edens JF, Lilienfeld SO, Cale EM (2003) Psychopathic personality or personalities? Exploring potential variants of psychopathy and their implications for risk assessment. Aggress Violent Beh 8:513–546. 10.1016/S1359-1789(02)00098-8 10.1016/S1359-1789(02)00098-8

[62] Tillem S, Chang S-AA, Baskin-Sommers A (2020) Comparison of socio-affective processing across subtypes of antisocial psychopathology. In: The Routledge handbook of the philosophy and science of punishment. Routledge, p 288–302

[63] Torres-Marín J, Navarro-Carrillo G, Carretero-Dios H (2022) Differentiating the traits of the Dark Tetrad in their linkages with humor styles, dispositions toward ridicule and laughter, and comic styles. Personality Individ Differ 185:111281. 10.1016/j.paid.2021.111281 10.1016/j.paid.2021.111281

[64] Drayton LA, Santos LR, Baskin-Sommers A (2018) Psychopaths fail to automatically take the perspective of others. Proc Natl Acad Sci 115:3302–3307. 10.1073/pnas.1721903115 29531085 10.1073/pnas.1721903115 PMC5879707

[65] Meffert H, Gazzola V, den Boer JA, Bartels AA, Keysers C (2013) Reduced spontaneous but relatively normal deliberate vicarious representations in psychopathy. Brain 136:2550–2562. 10.1093/brain/awt190 23884812 10.1093/brain/awt190 PMC3722356

[66] Wolf I, Dziobek I, Heekeren HR (2010) Neural correlates of social cognition in naturalistic settings: a model-free analysis approach. Neuroimage 49:894–904. 10.1016/j.neuroimage.2009.08.060 19733672 10.1016/j.neuroimage.2009.08.060

[67] Sharp C, Vanwoerden S, Van Baardewijk Y, Tackett JL, Stegge H (2015) Callous-unemotional traits are associated with deficits in recognizing complex emotions in preadolescent children. J Pers Disord 29:347–359. 10.1521/pedi_2014_28_161 25248014 10.1521/pedi_2014_28_161

[68] Winters DE, Sakai JT (2023) Affective theory of mind impairments underlying callous-unemotional traits and the role of cognitive control. Cogn Emot 37:696–713. 10.1080/02699931.2023.2195154 37017241 10.1080/02699931.2023.2195154 PMC10330116

[69] Chandler MJ (1973) Egocentrism and antisocial behavior: the assessment and training of social perspective-taking skills. Dev Psychol 9:326. 10.1037/h0034974 10.1037/h0034974

[70] Newbury-Helps J, Feigenbaum J, Fonagy P (2017) Offenders with antisocial personality disorder display more impairments in mentalizing. J Personal Disord 31:232–255. 10.1521/pedi_2016_30_246 10.1521/pedi_2016_30_246 27064853

[71] Bonta J, Andrews DA (2012) Viewing offender assessment and rehabilitation through the lens of the risk-needs-responsivity model. In: McNeil F, Raynor P, Trotter C (eds) Offender supervision: new directions in theory, research and practice. Willan, New York, pp 45–66

[72] Shepherd SM, Luebbers S, Ogloff JRP, Fullam R, Dolan M (2014) The predictive validity of risk assessment approaches for young Australian offenders. Psychiatry Psychol Law 21:801–817. 10.1080/13218719.2014.904262 10.1080/13218719.2014.904262

[73] Thompson AP, Putnins AL (2003) Risk-need assessment inventories for juvenile offenders in Australia. Psychiatry Psychol Law 10:324–333. 10.1375/pplt.2003.10.2.324 10.1375/pplt.2003.10.2.324

[74] Hawes DJ, Price MJ, Dadds MR (2014) Callous-unemotional traits and the treatment of conduct problems in childhood and adolescence: a comprehensive review. Clin Child Fam Psychol Rev 17:248–267. 10.1007/s10567-014-0167-1 24748077 10.1007/s10567-014-0167-1

[75] Jolliffe D, Farrington DP (2007) Examining the relationship between low empathy and self-reported offending. Leg Criminol Psychol 12:265–286. 10.1348/135532506X147413 10.1348/135532506X147413

[76] Hicks BM, Vaidyanathan U, Patrick CJ (2010) Validating female psychopathy subtypes: differences in personality, antisocial and violent behavior, substance abuse, trauma, and mental health. Personal Disord Theory Res Treat 1:38–57. 10.1037/a0018135 10.1037/a0018135 PMC2889701 20582155

[77] Dziobeck I, Fleck S, Kalbe E, Rogers K, Hassenstab J, Brand M, Kessler J, Woike JK, Wolf OT, Convit A (2006) Introducing MASC: a movie for the assessment of social cognition. J Autism Dev Disord 36:623–636. 10.1007/s10803-006-0107-0 16755332 10.1007/s10803-006-0107-0

